# Evaluation of tools for identifying large copy number variations from ultra-low-coverage whole-genome sequencing data

**DOI:** 10.1186/s12864-021-07686-z

**Published:** 2021-05-17

**Authors:** Johannes Smolander, Sofia Khan, Kalaimathy Singaravelu, Leni Kauko, Riikka J. Lund, Asta Laiho, Laura L. Elo

**Affiliations:** 1 Turku Bioscience Centre, University of Turku and Åbo Akademi University, 20520 Turku, Finland; 2 Institute of Biomedicine, University of Turku, 20520 Turku, Finland

**Keywords:** Copy number variation, Whole-genome sequencing, Ultra-low-coverage, Human embryonic stem cell

## Abstract

**Background:**

Detection of copy number variations (CNVs) from high-throughput next-generation whole-genome sequencing (WGS) data has become a widely used research method during the recent years. However, only a little is known about the applicability of the developed algorithms to ultra-low-coverage (0.0005–0.8×) data that is used in various research and clinical applications, such as digital karyotyping and single-cell CNV detection.

**Result:**

Here, the performance of six popular read-depth based CNV detection algorithms (BIC-seq2, Canvas, CNVnator, FREEC, HMMcopy, and QDNAseq) was studied using ultra-low-coverage WGS data. Real-world array- and karyotyping kit-based validation were used as a benchmark in the evaluation. Additionally, ultra-low-coverage WGS data was simulated to investigate the ability of the algorithms to identify CNVs in the sex chromosomes and the theoretical minimum coverage at which these tools can accurately function. Our results suggest that while all the methods were able to detect large CNVs, many methods were susceptible to producing false positives when smaller CNVs (< 2 Mbp) were detected. There was also significant variability in their ability to identify CNVs in the sex chromosomes. Overall, BIC-seq2 was found to be the best method in terms of statistical performance. However, its significant drawback was by far the slowest runtime among the methods (> 3 h) compared with FREEC (~ 3 min), which we considered the second-best method.

**Conclusions:**

Our comparative analysis demonstrates that CNV detection from ultra-low-coverage WGS data can be a highly accurate method for the detection of large copy number variations when their length is in millions of base pairs. These findings facilitate applications that utilize ultra-low-coverage CNV detection.

**Supplementary Information:**

The online version contains supplementary material available at 10.1186/s12864-021-07686-z.

## Background

Copy number variation (CNV) is defined as deletion or amplification of relatively large DNA segment (from 50 basepairs to several megabases) [1]. They contribute to genetic diversity and have relevance both evolutionarily and clinically. Massively parallel high-throughput DNA sequencing-based methods enable a rapid, cost-effective and flexible solution for the detection of genetic variants including CNVs. The advances in DNA sample and sequencing library preparation allows studying various sample types with limited amount of DNA-sample, e.g. in noninvasive detection of fetal aneuploidies from maternal plasma [2, 3], and in low-coverage detection of human genome variation [4, 5] as well as in the study of cancer-associated changes in cell-free plasma DNA [6–8]. In addition, the method provides a valuable tool to monitor chromosomal changes in *in vitro* cultured cells, including human embryonic stem cells (hESCs), which are known to accumulate genomic abnormalities during maintenance and expansion [9, 10]. Low-coverage sequencing is a valuable alternative for the cost efficient high-throughput monitoring of karyotypes of primary cell lines, such as human pluripotent cell lines, and is a necessity in order to karyotype formalin-fixed paraffin embedded (FFPE) samples [11, 12]. Low-coverage high-throughput single cell sequencing has also emerged in recent years and has been applied to study e.g. low-level mosaicism introduced by differing CNVs in cell subpopulations in cultured hESC samples [13]. In addition to the versatility of applications of low-coverage sequencing, the advantages of this approach also include lower costs and less computational resources and storage capacity compared to high-coverage sequencing.

Detection of CNVs from low and ultra-low-coverage sequencing data requires sensitive and reliable computational methods. Although many methods are available, their performance has so far been validated mainly on relatively high-coverage whole-genome sequencing (WGS) data (3–90×)[14–17]. Recently, the applicability of the CNV detection methods for noninvasive prenatal testing samples with read depth of 0.2–0.3× was assessed [18]. However, copy number profiling has been conducted from FFPE tumor samples with ultra-low read coverage 0.08× [12] and from cell-free DNA from tumor samples with ultra-low read coverage of 0.01× [19]. Presently, the ability of the methods to detect CNVs from such ultra-low-coverage sequencing data remains unclear.

To address this, we performed a systematic evaluation of six read depth based CNV detection algorithms, namely BIC-seq2 [20], Canvas [21], CNVnator [22], FREEC [23], HMMcopy [24], and QDNAseq [25] using ultra-low-coverage (0.0005–0.8×) WGS data. Read depth based algorithms in general are most suited to detect large CNVs also from low–coverage (≤ 10×) data, whereas other methodological approaches for CNV detection tend to require higher coverage; read pair, split read and assembly methods [18, 26]. We used both real-world WGS data with array-based and karyotyping based validated CNVs as well as simulated CNVs as benchmarking data. Compared to array-based and karyotyping based benchmarking data, simulated CNVs provide the most accurate ground truth in respect to exact breakpoints of the CNVs. Simulated data also allowed us to investigate multiple CNVs of different sizes simultaneously and include benchmark CNVs in the X and Y chromosomes. Sex chromosomes have been shown to harbor CNVs of evolutionary and clinical interest [27–29] and thus tools’ ability to call CNVs in the sex chromosomes besides the autosomes were evaluated. The computational demand was assessed by running time, memory requirement and failure rate.

## Results

**Fig. 1:**
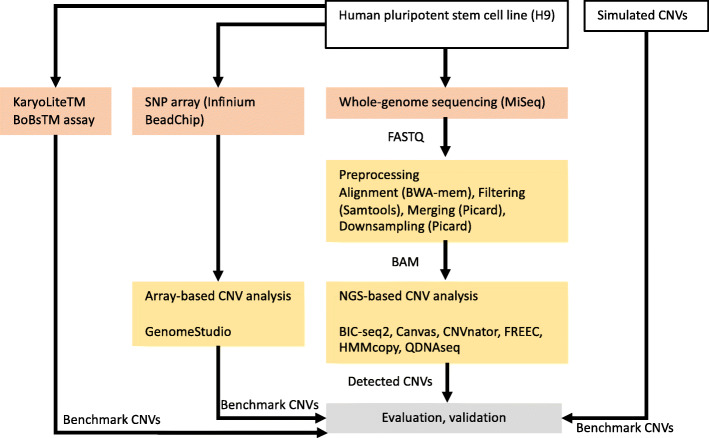
Flowchart showing the main steps of our comparison, including preprocessing of the data, detection of copy number variations (CNVs) with six different algorithms (BIC-seq2, Canvas, CNVnator, FREEC, HMMcopy, and QDNAseq) and evaluation and validation of the results. The karyotyping results from the KaryoLiteTM BoBsTM assay are from an earlier study [9]

In this section, we describe the results of the comparison of six CNV detection tools (BIC-seq2, Canvas, CNVnator, FREEC, HMMcopy, QDNAseq), which are summarized in Table 1 and discussed further in Methods section. In the first part of this section, we benchmark the methods using simulated WGS data, which enables us to study simultaneous deletions and duplications in autosomal and sex chromosomes. In addition, we obtain information about the optimal window size for each method at different read coverages (0.0005–0.8×). We utilize the optimal window size information in the second part of this section, where we benchmark the methods using real hESC cell line data and evaluate the results using microarray and karyotyping kit-based data. In both parts of the comparison, we measure the performance using sensitivity, false discovery rate (FDR) and F1 score. Finally, we also compare run times of the methods. Figure 1 illustrates the mains steps of the comparison process.

**Table 1:** Summary of features for the algorithms

Feature	BIC-seq2	Canvas	CNVnator	FREEC	HMMcopy	QDNAseq
Language	C++, Perl, R	C#	C++	C++, R	C++, R	R
Input format	BAM	BAM	BAM	BAM, many other	BAM	BAM
Control sample	optional	optional	no	optional	optional	yes
User-defined/built-in window size	built-in	built-in	user	both	user	user
Fixed window size	yes	no	yes	yes	yes	yes
GC-correction	yes	yes	yes	yes	yes	yes
Mappability correction	yes	no	no	yes	yes	yes
Sex-determination	From XY CNVs	From XY CNVs	From XY CNVs	User-specified	From XY CNVs	From XY CNVs. By default, XY excluded.
Segmentation	BIC^1^	Haar wavelet (default), CBS^2^	Mean shift	LASSO^3^	HMM^4^	CBS^2^
Version	0.2.4, 0.7.2	1.11.0	0.3.3	11.0	1.20.0	1.14.0
Reference	[20]	[21]	[22]	[23]	[24]	[25]

^1^Bayesian information criterion

^2^circular binary segmentation

^3^least absolute shrinkage and selection operator

^4^hidden Markov model

### CNV algorithm evaluation using simulated data

**Fig. 2:**
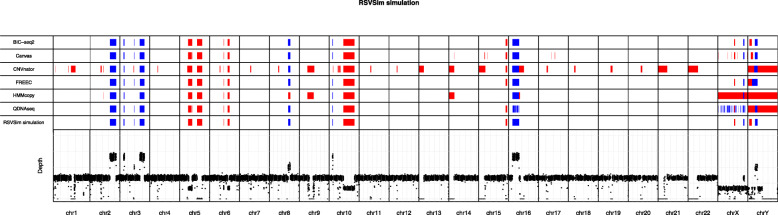
Genomic map visualization of the copy number variations (CNVs) detected in the simulated dataset using the six algorithms (rows 1–6) along with the ground truth CNVs (row 7) in the respective chromosomal locations. Deletions are marked in red and duplications in blue. The bottom part of the visualization depicts the depth of read coverage at each 50 kbp window. The read coverage of the data used in this visualization was 1×

In total nine deletions and nine duplications of ≥ 1 Mbp were generated as benchmark CNVs in the simulated WGS data (Supplementary Table 1). The genomic map in Fig. 2 visualizes the CNVs predicted by all six algorithms along with the simulated ground truth CNVs in all 24 main human chromosomes. With the coverage of 1×, FREEC and BIC-seq2 were able to accurately detect all 14 CNV regions (seven duplications and seven deletions) in autosomes without any false positive detections. Canvas and QDNAseq also detected correctly all the autosomal CNVs, but Canvas produced also some additional false positives, whereas QDNAseq produced some copy number neutral segments within some of the CNVs. HMMcopy failed to identify a small 1 Mbp duplication in the chromosome 3. Two of the tools predicted the correct location, but a false copy number for some of the CNVs; CNVnator reported the duplication in the chromosome 10 as deletion, and HMMcopy reported the duplication in the chromosome 8 as deletion. In addition, unlike the other methods, CNVnator was not able to discard centromeres as problematic regions, and it instead reported them as homozygous deletions.

The simulated benchmark data included two 5 Mbp CNVs (one deletion and one duplication) in the X and Y sex chromosomes. The results show that only BIC-seq2 was able to accurately detect all of the CNVs in both sex chromosomes, whereas the other tools had more or less difficulties in predicting them. BIC-seq2 was the only algorithm that was able to accurately detect both of the CNVs in the chromosome Y. While Canvas correctly identified the duplication in the chromosome Y, it mislocated the deletion. FREEC reported larger segments for the deletion and for the duplication without a copy number neutral region between the two CNVs. All of the algorithms, except HMMcopy, were able to detect the duplication in the chromosome Y. BIC-seq2, Canvas and FREEC were able to detect the CNVs in the chromosome X correctly. HMMcopy was able to detect the duplication correctly in the chromosome X, but failed to detect the deletion, and it instead reported a large deletion spanning almost the entire chromosome. CNVnator did not report any CNVs in the chromosome X, whereas QDNAseq predicted several small CNVs.

**Fig. 3:**
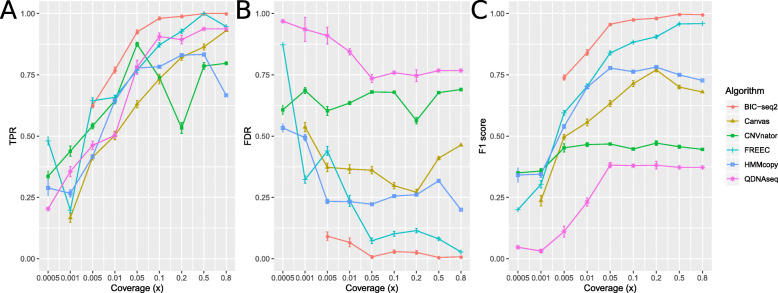
Performance evaluation of the six copy number variation (CNV) algorithms using the simulated data with the stringent criteria: at least 80 % overlap between the inferred and ground truth CNVs and no filtering by CNV length. **a** True positive rate (TPR), **b** False discovery rate (FDR), and **c** F1 score of the CNV detections achieved by the different tools when the read coverage is varied. For each algorithm and coverage, the data point values depict the performance values achieved using the window setting that provided the highest F1 score (Supplementary Figs. 2, 3, 4, 5, 6). Error bars denote the standard error of the results generated from the results of 20 different random subsets

In order to assess how the coverage of the simulated WGS data affects the performance, we used nine different coverages (0.8×, 0.5×, 0.2×, 0.1×, 0.05×, 0.01×, 0.005×, 0.001×, and 0.0005×). The original simulated dataset with coverage of 1× was downsampled to each of the nine different coverages 20 times. The average sensitivity, FDR and F1 score of the six CNV algorithms were calculated using stringent (≥ 80 % CNV segment overlap) and loose (≥ 60 % CNV segment overlap and inclusion of only ≥ 0.5 Mbp CNV segments) criteria, as shown in Fig. 3 and Supplementary Fig. 1, respectively. Overall, the choice of the evaluation criteria had no effect on the order of the best and poor-performing tools, and there was not considerable variation in the inferred CNVs for any of the tools across the twenty subsets of the data.

In general, when using either the stringent or loose criteria all of the tools performed poorly with extremely low read coverages (0.0005× to 0.01×) and better with higher coverages. All of the tools achieved ≥ 50 % sensitivity with read coverages ≥ 0.01x. BIC-seq2 outperformed the other tools with the lowest FDR values and the best sensitivity and F1 scores (≥ 0.05×), followed by FREEC. BIC-seq2 worked well even with a read coverage of as low as 0.005×, which corresponds to only 50 000 read pairs, achieving a relatively high F1 score of 0.75, but failed to complete the analysis with the lower coverages. CNVnator produced a lot of false positive detections, resulting in a lower than average general performance (highest FDR in ≥ 0.001× read coverages and lowest F1 score in ≥ 0.005× read coverages) (Figs. 2 and 3 and Supplementary Fig. 1). However, CNVnator achieved high sensitivity with many of the window sizes (Supplementary Fig. 2), when not considering the results in the F1 score optimized way as in Fig. 2. The false positives are mainly attributable to the centromere regions that CNVnator was not able to exclude. Canvas benefitted from the looser criteria (Supplementary Fig. 1) and was then noticeably closer to the performances of FREEC and BIC-seq with all the coverages.

Next, five different window sizes (100, 200, 500, 1000, and 2000 kbp) were tested to investigate the relationship between the coverage and the optimal choice of the window size. Canvas was not considered in the window size comparison, as it works by a different approach based on fixing the number of reads per window. The results of these comparisons are shown in Supplementary Figs. 2, 3, 4, 5, 6. The results suggested that with each method the window size had a considerable effect on the performance and the methods responded differently to its adjustment. For example, changing the window size from 100 to 2000 affected the performance of BIC-seq2 noticeably in higher coverages (0.05–0.8×), decreasing the sensitivity and increasing the FDR. For CNVnator, on the other hand, a smaller window size improved the sensitivity, but increased the FDR. We used the F1 values of the window size comparison to select the optimal window size for each method at coverage of 0.1×, which we used in the cell line data benchmarking. It should be noted that some of the larger windows sizes (1 Mbp, 2 Mbp) were likely too large for the identification of the smallest CNVs of 1 Mbp length. However, this is not an issue that affects the method comparison, as the same window size was optimized for each coverage and method before the comparison.

### CNV algorithm evaluation using cell line data

The real WGS data were from karyotypically normal (H9-NO) and abnormal (H9-AB) variants of the hESC cell line H9, harvested for the analysis at different passages of 38 and 41 (H9-NO-p38 and H9-NO-p41) or 113 and 116 (H9-AB-p113 and H9-AB-p116); Supplementary Table 2. The CNVs detected in the SNP array validation data were used as benchmark CNVs; the CNVs ≥ 500kbp are described in detail in Supplementary Table 3. In normal cell line samples (H9-NO-p38 and H9-NO-p41), only one gain (in chromosome 7) was detected using the SNP array data. This same gain was also present in the abnormal samples (H9-AB-p113 and H9-AB-p116), with additional gains in the chromosomes 17 and 20. In the chromosome 12 there were two gains separated by a centromere in H9-AB-p116, whereas in H9-AB-p113 the chromosome 12 gain was fragmented into four segments (Supplementary Table 3).

**Fig. 4:**
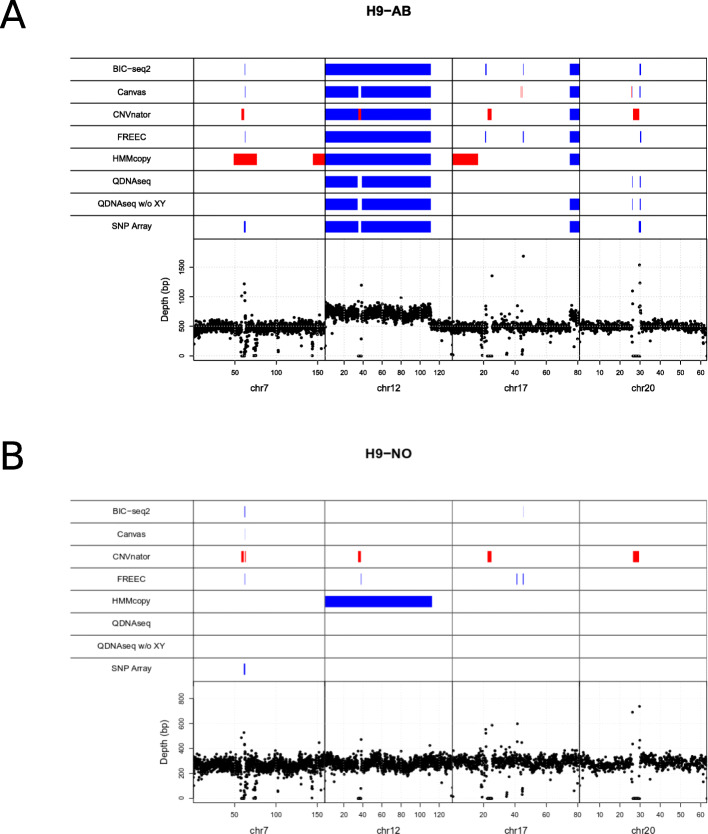
Visualization of the CNVs detected in the cell line data with the six algorithms along with the array-based benchmark CNVsin the respective chromosomal locations. **a** Karyotypically abnormal (H9-AB) and **b** normal (H9-NO) variants of the human embryonic stem cell line H9 were analysed. Deletions are marked in red and gains in blue. The bottom part of the visualization depicts the depth of read coverage at each 50 kbp window

Figure 4 a and b show genomic map visualizations for the combined abnormal and normal samples, respectively, which include the benchmark CNVs ≥ 500 kbp and the predicted CNVs by each method. The same visualization is available for the individual samples in Supplementary Figs. 7, 8, 9, 10. For QDNAseq the CNV detection is visualized using two different setups: inclusion and exclusion of the sex chromosomes X and Y. BIC-seq2, Canvas and FREEC are the only algorithms that found the gains in chromosomes 7 and 20. However, none of the tools met the minimum overlap criterion of > = 80 %. All of the algorithms found the large chromosome 12 gain. The fragmented detection of QDNAseq and Canvas for the chromosome 12 gain can be explained by the exclusion of the blacklisted regions that both algorithms use by default. In order to further evaluate the tools’ performance, we examined the detection accuracy genome-wide, i.e. including all the chromosomes for combined abnormal sample and combined normal sample (Supplementary Figs. 11 and 12, respectively) and for the individual samples separately (Supplementary Figs. 13, 14, 15, 16). With these combined samples all the tools report varying amount of false positive detections, with largest number of false positives reported by HMMcopy.

**Fig. 5:**
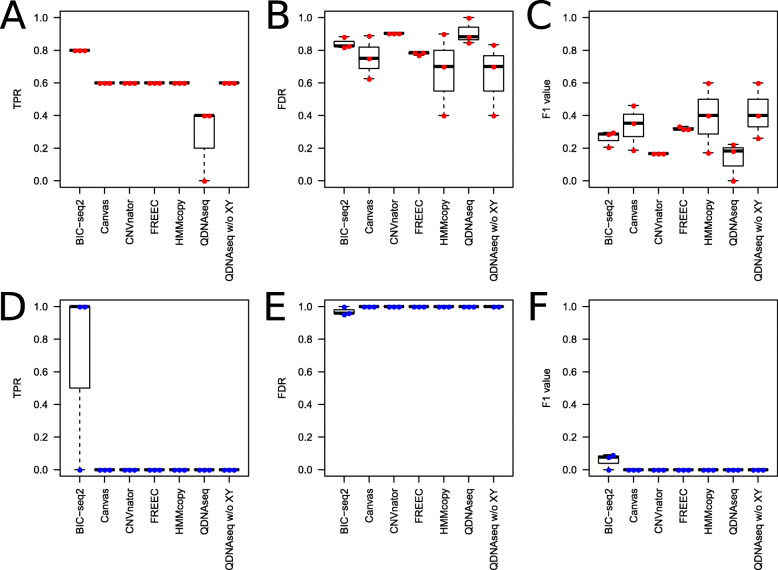
Performance evaluation of the six algorithms using the cell line data with the criteria of ≥ 50 % overlap and no minimum length requirement for the detected CNVs. **a**, **d** True positive rate (TPR), **b**, **e** False discovery rate (FDR), and **c**, **f** F1 score of the CNV detections. The red and blue dots depict the abnormal and normal samples, respectively

We calculated the sensitivity, FDR and the F1 score for the results of each algorithm using the real-world cell line data and less stringent criteria compared to the simulated data: the CNV overlap was required to be ≥ 50 % and no length requirement for the detected CNV was set (Fig. 5). In this setting, most of the algorithms detected the gain in the chromosomes 12 and 17 of the abnormal samples, and hence the sensitivity of the algorithms was similar (Fig. 5 a). BIC-seq2 had clearly the best sensitivity with both the abnormal and normal data, because BIC-seq2 was able to identify also some of the smaller gains in the chromosomes 7 and 20. However, the loose criteria increased drastically the number false positive with all the methods, producing universally high FDR values and low F1 scores. In general, the FDR results for the six tools were in accordance with the results obtained from the simulated data. Here as well BIC-seq2 and FREEC reported fewer false positives, whereas CNVnator and QDNAseq had the highest average FDR. However, QDNAseq achieved without the sex chromosomes the lowest average FDR for the abnormal data.

In addition, we inspected the performance using more stringent criteria of ≥ 80 % CNVoverlap and at least 500 kbp CNV length requirement for the detected CNVs. With these stringent criteria none of the algorithms detected the only gain in the normal samples (Supplementary Fig. 17). With the length requirement of at least 500 kbp we found QDNA-seq without the sex chromosomes to be the best tool, achieving the lowest and highest average FDR and F1 score, respectively, followed by BIC-seq2 and Canvas.

All the algorithms were run with the sex chromosomes included. Additionally, QDNAseq was run separately without the sex chromosomes, because QDNAseq excludes the sex chromosomes by default. The analysis of the simulated data showed that QDNAseq achieved one of the best sensitivities in the comparison (Fig. 1 and Supplementary Fig. 1). However, with the real cell line data the sensitivity or QDNAseq was considerably lower when the sex chromosomes were included compared to when they were not included.

**Fig. 6:**
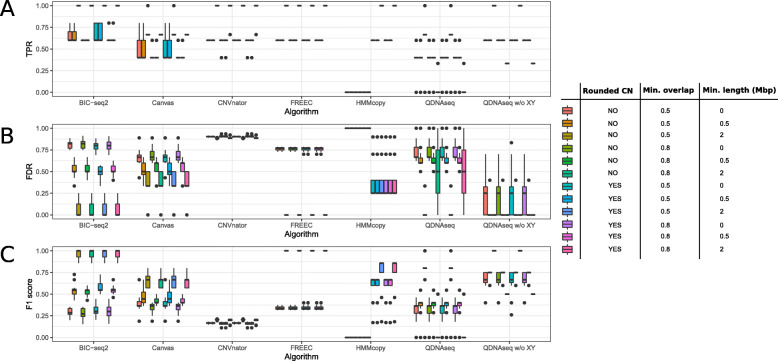
Performance evaluation of the six algorithms using the combined abnormal cell line samples while varying three evaluation parameters: rounded copy number (CN) value (yes or no), minimum overlap (50 or 80 %) and minimum CNV length (no restriction (0), ≥ 0.5 Mbp or ≥ 2 Mbp). **a** True positive rate (TPR), **b** False discovery rate (FDR), and **c** F1 score of the CNV detections for each of the six tools

The results that we discussed above were calculated using rounded copy number values, i.e. no distinction between homozygous and heterozygous CNVs was made. Moreover, the small gains in the chromosome 7 and 20 might be spurious, and we wanted to focus on the larger CNVs, which is why we also discarded the normal samples and included only the abnormal samples for the next step. We compared the methods further by varying three evaluation parameters (Fig. 6): rounded copy number value (yes or no), minimum overlap (50 or 80 %), and minimum CNV length (no restriction (0), ≥ 0.5 Mbp or ≥ 2 Mbp). When evaluating the CNVs by their exact copy number, no impact on the sensitivity, FDR or the F1 score was observed for five of the six tools, HMMcopy being the only exception. With the loosest criteria (50 % overlap and ≥ 2 Mbp length), FREEC and QDNAseq without the sex chromosomes were the best-performing methods based on the F1 scores. Unlike QDNAseq, FREEC was also able to achieve perfect average F1 score with the overlap of 80 %, which is why we considered it the best method of the cell line benchmarking. BIC-seq2 found some false positives, which is why it was slightly worse than the two methods. As in the simulation, CNVnator produced a high number of false positives, which is again mainly attributable to the false homozygous deletions in the centromere regions. Canvas achieved the lowest average sensitivity among the methods and moderate FDR, explaining the lower F1 scores.

The CNV detection methods have differences in how they handle the centromeres, affecting the evaluation of the large gain in the chromosome 12. The SNP array, Canvas and QDNAseq predicted that there was a copy number neutral gap in the centromere region, whereas FREEC, BIC-seq2 and HMMcopy identified the gain as one complete segment spanning across the centromere. Our approach was to treat the SNP array as the ground truth and no changes were made to its CNV list besides the size filtering. The real CNV might actually follow the whole CNV structure and not the segmented structure, which is why the wrong methods might be penalized for the centromere. However, this was not a significant issue in our comparison due to the small size of the centromere and our comparison approach that penalized for the redundant segmentation based on the size of the gaps.

Finally, we compared our results to the previous karyotyping experiment with KaryoLiteTM BoBsTM assay [9]. That experiment found only a single large gain in the chromosome 12 for the H9 cell line, which corresponds to the same gain detected using both the SNP array data and all six algorithms.

### Running time, memory requirement and failure rate

A computer cluster node with 16 Intel(R) Xeon(R) CPU E5-2670 at 2.60GHz cores and 64 GB of random-access memory (RAM) was used to perform the analyses in this study. All the algorithms were run using 20 GB of RAM. If the algorithm workflow included transforming alignment BAM files into other formats (e.g. hits or wig), then the time used for this was included in the total running time. We measured the running time for each algorithm while running the four cell line samples (H9-AB-p113, H9-AB-p116, H9-NO-p38 and H9-NO-p41) with the same parameters as were used in the evaluation.

There were considerable differences in the running times, HMMcopy being clearly the fastest algorithm and BIC-seq2 the slowest (Table 2). The slowness of BIC-seq2 is attributable to the computationally demanding normalization step, accounting for 99.9 % of the run time. In terms of the real maximum memory consumption, FREEC and Canvas were the lowest and highest memory consumers, respectively.

**Table 2:** Mean and standard deviation (SD) of the running times in seconds and maximum memory consumption for each algorithm

Algorithm	Running time (s)	Max RSS (MB)
BIC-seq2	8389 ± 1222	3753 ± 0
Canvas	764 ± 9	10,933 ± 16
CNVnator	543 ± 2	7288 ± 1
FREEC	168 ± 2	1 ± 0
HMMcopy	69 ± 5	99 ± 0
QDNAseq	105 ± 1	287 ± 50

The failure rates at each coverage was estimated by calculating the proportion of the runs in the simulation experiment that failed to complete. All of the algorithms had zero failure rate with read coverages ≥ 0.01x. With lower coverages BIC-seq2 was the least stable algorithm, followed by Canvas and then FREEC. (Supplementary Table 4). We investigated the error messages to try to discover what caused the failure of each method. BIC-seq2 failed during fitting the Generalized Additive Model using the mgvc R package, because there was “not enough data to do anything meaningful”, suggesting that it was not designed for coverages that low. For Canvas we were unable to find a potential cause, but the issue was that it generated empty VCF files with only headers. This is unusual behavior by Canvas, because normally the VCF file includes also the copy number neutral segments. Regarding FREEC, its error was related to fitting the linear regression and the expectation-maximization models.

## Discussion

We have performed a comparative analysis to evaluate the performance of six CNV detection algorithms (BIC-seq2, Canvas, CNVnator, FREEC, HMMcopy, QDNAseq) using ultra-low-coverage (0.0005-0.8x) WGS data. These tools were selected because they are commonly used either based on the number of citations or the number of downloads of the tool. We only selected one tool as representative when several tools work under a similar functioning principle, e.g. QDNAseq and CNAnorm [30] both utilize circular binary segmentation in window segmentation. Furthermore, we included only the tools that do not require an external control sample and thus did not include tools such as CNV-seq [31], VarScan2 [32], TitanCNA [33] or WisecondorX [18]. From the selected tools BIC-seq2, FREEC, HMMcopy and Canvas can be optionally used with a control sample to produce a copy number ratio between the test and control samples, whereas CNVnator only processes individual samples against the reference genome and QDNAseq automatically includes a general control sample (bin annotation).

All of the selected tools were read depth based algorithms. They all share a very similar workflow, consisting of four main steps. In the first step read alignments were counted within genomic windows (or bins) of a certain size that were either selected by the user or dynamically-determined by the algorithm. Some algorithms (here only Canvas), however, fix the number of read alignments per window instead, meaning the window size itself varies. In the second step, systematic biases from the counts are removed. The two principal causes of systematic biases in the read alignment efficiency are the local GC-content and mappability of the different genomic regions [34]. Some methods aim to account for these by using the reference genome to determine how much each window is likely to be affected by these factors and then adjusting the window-wise count values accordingly. In the third step, segmentation of the counts into homogeneous regions with highly-similar copy numbers is performed. Segmentation is typically performed by advanced machine learning, signal processing or statistical methods that are used to infer which windows are part of the same CNV. Additionally, Canvas and QDNAseq used blacklisting to filter out problematic regions. In the final step, a copy number value was assigned to each segment. Some algorithms (e.g. Canvas) also generated a confidence estimate or *p*-value for each CNV, typically by testing whether mean read depth or ratio of a segment differed from the genomic average. All the six algorithms were also coupled with convenient visualization functions that can be used in illustrating the effect of bias correction or in the interpretation of the results.

Based on our evaluation, BIC-seq2 and FREEC were the two best-performing tools. With the cell line data FREEC was the best algorithm with only a narrow marginal to the next best-performing tool (BIC-seq2). However, BIC-seq2 outperformed all other tools with the simulated data and was the only algorithm that could accurately detect CNVs in the sex chromosomes. Both FREEC and BIC-seq2 performed well even on extremely low coverages (0.01-0.1x) in the simulated data. The varying window size affected BIC-seq2 performance more than FREEC, but both tools performed better when using short (100 bp to 200 kbp) rather than long window sizes. Since BIC-seq2 is run in two steps, with the normalization step being considerably slow, it was clearly the slowest of the six tools.

Many methods produced strikingly large numbers of false positive detections especially when smaller CNVs were detected. On the other hand, both the sensitivity and FDR improved when assessing the detection of large CNVs (length being millions of base pairs). This is in line with previous studies that have concluded that large CNV detection from 1× coverage WGS data is an efficient approach and even outperforms array-based CNV analysis [35]. It should be noted that for some of the methods, the number of false positives can be potentially decreased by improving the filtering that excludes problematic regions. CNVnator would considerably benefit from blacklisting centromere regions, whereas QDNAseq would benefit from disabling some of the filters that cause copy number neutral gaps to the CNVs in the autosomes and false positives to the sex chromosomes.

CNVs in sex chromosomes have many clinical implications. Surprisingly large variability was observed in the algorithms’ ability to identify CNVs in the sex chromosomes X and Y. BIC-seq2 accurately detected the two simulated CNVs in sex chromosomes and thus makes it suitable for karyotyping and other applications where the ability to detect CNV regions in chromosomes X and Y is important.

HMMcopy performed well in a previous comparative study, where somatic CNVs were detected from both simulation and primary tumor data [15], ranking fourth in the simulation data and first in the primary tumor data. In our analysis, HMMcopy managed to detect the two largest gains without producing excess false positive detections. It was also the fastest to run among the compared algorithms. However, HMMcopy did not separate well between homozygote or heterozygote CNVs. Another study found CNVnator to perform best [36]. In our analysis CNVnator was able to detect the two largest array-validated duplications, but many additional unvalidated deletions were simultaneously detected. When using simulated data, the algorithm was among the worst-performing tools due to the high number of false positives. However, the sensitivity of CNVnator improved with high-coverage data (0.8×). Both of the previous studies have used much higher coverage than was used here and thus CNVnator may be more suitable for high-coverage data. Canvas performed well in a previous comparative analysis where data with much higher coverage (40–80×) were used [21]. Here, Canvas started to raise errors with the lower coverages. Canvas was accurate with the simulated data when small CNVs were filtered out (Supplementary Fig. 1), but with the cell line data its performance was moderate.

We acknowledge that the simulated data we generated was not perfectly realistic. This might cause issues in the comparison, because many methods correct the data for the local GC content and mappability. The varying mappability is taken into account to some degree, which is visible from the genomic map visualization where regions with lower read depth are present near the centromeres (Fig. 2). However, the impact of these confounding factors decreases when we consider larger CNVs with larger window sizes. It should also be noted that many of the methods that apply the corrections still performed excellently with the simulated data, such as FREEC and BIC-seq2. However, we cannot rule out the possibility that some of the tools would benefit from more optimally simulated data.

It is also important to mention that some of the methods considered in this comparison were not originally designed for germline CNV detection. The results suggested that the tools that were designed for tumor samples (HMMcopy and QDNAseq) exhibited, on average, inferior performance. However, we included them into our comparison, because they were designed for detecting large CNVs and they can, according to the developers, still be used with other sample types as well. Although QDNAseq achieved high sensitivity with the simulated data, its sensitivity decreased for the real cell line data when the sex chromosomes were included. This could be attributable to the CGHcall component of QDNAseq, which was originally designed for array data. According to the developers, CGHcall also benefits from analyzing multiple samples simultaneously, but this is not always practical in CNV detection where experiments with only one sample are not uncommon.

A common challenge in bioinformatics methods comparisons is that the methods have often many hyperparameters, whose tuning can change the results. Here, we compared the methods using their default parameters and recommendations given by the developers, because this is the most common approach by the users. We observed that for most parameters there were no instructions on how to adjust them for a certain sequencing read depth. However, the window size was considered an important parameter in the present work, as it directly affects the size of the CNVs that can be identified. For this reason, we adjusted the window size for each method that has no automated method for optimizing the window size for a specific read depth. Although it could potentially provide new interesting insights into the methods, if their different parameters were optimized in a comprehensive manner, addressing this was beyond the scope of this study.

Furthermore, many of the tools were not readily usable with our 2 × 150 bp sequencing setup (BIC-seq2, FREEC, HMMcopy and QDNAseq). This required us to generate a new mappability track using GEMtools, instead of being able to use the default tracks provided by the tools. The user can also circumvent this by trimming the raw reads shorter using tools like Trimmomatic, which is what we did for QDNAseq, because its bin annotation needs to be generated based on a set of multiple control samples with the same read chemistry. However, the drawback of this approach is that it decreases the accuracy of the read alignment, and hence it could decrease the accuracy of the CNV detection.

In addition to the detection of large CNVs in hESCs, accurate detection of large CNVs from ultra-low-coverage WGS data can have many other potential applications, e.g. in prenatal diagnostics. However, the suitability and performance of the CNV detection methods coupled with ultra-low-coverage WGS in the other application fields, such as identifying sex chromosome anomalies, requires further studies.

## Methods

The main steps of our comparison approach are shown in Fig. 1 and described here in more detail.

### Data sets

In this work, we used simulated WGS data as well as WGS data from hESC samples to evaluate the performance of the CNV detection tools.

### Simulated data

To investigate the ability of the algorithms to identify CNVs in sex chromosomes, and to also acquire a more genuine ground truth for the purpose of benchmarking, we created simulated WGS data. The RSVSim v1.18.0 was used to create a FASTA reference (Hg19) with the CNVs, followed by wgsim v1.6 to generate 2 × 150 length short reads using the reference. We generated 10 million read pairs with wgsim, which equals to 1x depth of coverage (before filtering). We aligned the reads to the simulated FASTA reference using BWA-mem v0.7.16a and extracted reads mapped in proper pairs and with mapping quality of at least 30. We included all autosomal and sex chromosomes into this simulated dataset. We did not differentiate between homozygous and heterozygous CNVs, but considered only two types of CNVs, deletions and duplications, in our evaluation (Supplementary Table 1).

### Human pluripotent embryonic stem cell line H9 sample preparation

Karyotypically normal (H9.N) and abnormal (H9.AB) sublines of human pluripotent embryonic stem cell line H9 from WiCell Research Institute, Inc [37] were used in this study. These hESC lines were expanded for the experiments on Matrigel-coated cell culture plates in mTeSR1 medium (Stem Cell Technologies) as previously described [38, 39]. The H9 cells with normal karyotype were harvested for the analysis at passages 38 and 41 and karyotypically-abnormal cells at passages 113 and 116. The cells were lysed in Qiagen RLT buffer by passing through syringe and 21G needle for five times. The genomic DNA was isolated with Qiagen Allprep miRNA/RNA/DNA Universal kit according to manufacturer’s instructions. The quality and quantity of the DNA was analyzed with Nanodrop and Qubit 2.0, and fragment size determined with 2 % SYBR Safe E-gel (all from Thermo Fisher Scientific).

### Library preparation and low-coverage sequencing

The samples were prepared for sequencing in two technical replicates. One nanogram of genomic DNA was used as a starting material for the library preparation. The libraries were prepared with Illumina Nextera XT DNA kit according to manufacturer’s instructions. The quality of the libraries was determined with Agilent 2100 Bioanalyzer. The libraries were sequenced in one flow cell with Illumina MiSeq Next-Generation Sequencer with 2 × 150 bp chemistry.

### Sequencing data processing

Quality control of the raw sequence data was performed using FastQC v0.11.4 (https://www.bioinformatics.babraham.ac.uk/projects/fastqc/). Alignment of the reads was done with BWA-mem v0.7.16a [40] against the human reference genome hg19. We used the older reference, because it is more commonly supported by CNV detection tools (e.g. QDNAseq). The uniquely-aligned reads were extracted for the downstream analysis with SAMtools v1.6 [41].

The replicate samples (four replicate samples for the abnormal samples H9-AB-p113 and H9-AB-p116, and two replicate samples for normal H9-NO-p38 and H9-NO-p41) were analyzed both individually and as combined. In the combined samples the BAM files were merged as follows: replicates 1 and 2 were merged resulting in four samples H9-AB-p113, H9-AB-p116, H9-NO-p38 and H9-NO-p41. Finally, all the eight replicate samples for H9-AB and four replicate samples for H9-NO were combined as one sample, respectively (Supplementary Table 2). Read coverages were calculated according to the Lander/Waterman equation [42] based on the number of bases, which yielded 0.67x coverage for the abnormal sample and 0.36x coverage for the normal sample (Supplementary Table 2).

### Karyotyping and SNP microarray-based validation

The number and shape of chromosomes of the samples were determined, i.e. karyotyped, using G-banding and KaryoLiteTM BoBsTM (Perkin Elmer) methods [9]. The karyotypes were validated with Illumina Infinium CoreExome-24 v1.1 BeadChip according to the manufacturer’s instructions.

The genotyping data were analyzed using Illumina’s GenomeStudio v2.0 software and its CNV Analysis Plugin was used to detect the CNVs for each sample separately. The software detects the CNVs based on the relative intensity shifts between breakpoints along the chromosomal segments, and the cnvPartition algorithm is used to calculate the copy numbers and their associated confidence scores [43]. The CNVs of ≥ 500 kb were included in a benchmarking dataset (Supplementary Table 3).

### CNV detection algorithms

We selected six popular CNV detection algorithms for our comparison, namely BIC-seq2, Canvas, CNVnator, FREEC, HMMcopy, and QDNAsEq. Below, we give a brief overview of each CNV detection algorithm used in this study, with key features summarized in Table 1.

BIC-seq2 has two main parts that are ran separately, namely BIC-seq2-norm and BIC-seq2-sEq. BIC-seq2-norm performs the mappability and GC-content corrections at single base level. In the BIC-seq2-seq part, the Bayesian information criterion (BIC)-based segmentation is performed where similar neighboring bin pairs are merged in an iterative fashion. The default bin size is 100 bp and can be adjusted by the user.

Canvas is developed by the sequencing instrument manufacturer Illumina, Inc. and it is included in the company’s Isaac whole-genome sequencing workflow. In addition to the germline WGS workflow that we employed, the tool also supports three other modes: somatic CNV analysis based on WGS data and tumor/normal sample pair analysis of targeted sequencing data. Both GC-content correction and mappability correction are supported. Instead of selecting a fixed window size, the windows are generated based on a number of read alignments per window (default is 100), which leads to variable-sized windows. Haar wavelet segmentation is used by default, but circular binary segmentation (CBS) is also supported. Loss of heterozygosity (LOH) regions are reported along with CNVs.

CNVnator functions without control sample. GC-correction is available, while mappability correction is not supported. The window size is determined by the user and segmentation is based on the mean-shift technique.

FREEC is a tool that can be used to detect CNVs, but also LOH regions from whole-genome sequencing (WGS) or whole-exome sequencing (WES) data. A control sample is required for WES data, but is optional for WGS data. GC-content and mappability correction are recommended when no control sample is used. Window size can be set by the user or determined dynamically by the algorithm. A least absolute shrinkage and selection operator (LASSO)-based algorithm is used for the segmentation.

HMMcopy supports CNV analysis with and without control sample and the window size needs to be set by the user. Both GC-content and mappability correction are supported, but not strictly required. A hidden Markov model (HMM) based approach is used for the segmentation and copy number assignment. HMMcopy is described as a CNA detection tool for tumour samples, but it is also applicable to other sample types.

QDNAseq is different compared to the others in the sense that it requires control data. The control data are used to generate the bin annotation, which is specific to certain window size, read length and reference genome. The bin annotations are used to correct for errors in the GC content and the mappability. The user can either download already available bin annotations that were generated based on a a set of control samples or generated own. CBS algorithm is applied for segmentation and the identification of the abnormal copy number regions. The performance of the method has been previously demonstrated on low-coverage data (0.1x) [25].

We used the default parameters for all the algorithms with two exceptions. First, since QDNAseq does not include sex chromosomes by default, we ran it with and without sex chromosomes. The analysis with sex chromosomes was performed as instructed in the R package manual of QDNA-sEq. Second, since half of the algorithms (CNVnator, QDNAseq, HMMcopy) have no default value for the window size and since the window size can be altered for all the tools except for Canvas, we investigated how the choice of the window size affected the performance. We tested five different window sizes (100, 200, 500, 1000, and 2000 kbp) when analyzing the simulated data. However, for QDNAseq we tested different window sizes that were avaible in the bin annotation of the R package (50, 100, 500 and 1000 kbp). Additionally, since FREEC can also adjust the window size automatically based on the coverage, and BIC-seq2 has a default value (100 bp), we also included their results into our comparison.

### Algorithm evaluation

In benchmarking, we used three statistical measures: sensitivity, i.e. TPR, false discovery rate (FDR) and F1 score. The true positive (TP) and false negative (FN) CNV detections were defined by comparing the ground truth CNVs against the inferred CNVs. First, for every CNV in the ground truth we searched all CNVs with the same copy number in the inferred CNV list that overlapped the ground truth CNV by at least one base. Next, we calculated the ratio of how many bases the two genomic region sets overlapped to the length of the ground truth CNV. A threshold was set for this quantity to classify the CNV as either TP or FN, which was set to 60 and 80 % for loose and stringent criteria, respectively, for the simulated data. Every inferred CNV that did not overlap with any of the ground truth CNVs were counted as false positive (FP). In addition to the requirement for minimum overlap we filtered CNVs based on their length. CVNs that were shorter than 0.5 Mbp (rounded) were not considered. The results for each algorithm were visualized in a genome map to see how well the inferred and ground truth CNVs are in agreement. To generate the read-depth-per-window counts for the visualizations, we used BEDTools v2.17.0 [44].

To investigate how the coverage in combination with the different window sizes affected the results, we tested nine different coverages (0.8x, 0.5x, 0.2x, 0.1x, 0.05x, 0.01x, 0.005x, 0.001x, and 0.0005x) by downsampling simulated BAM file with Picard’s DownSampleSam function [45]. To account for randomness in downsampling we generated 20 different random subsets for each coverage. For each algorithm and combination of coverage and window size the sensitivity, FDR and F1 score were calculated.

When the mappability tracks matching to our read chemistry (2 × 150 bp) were not publicly available, the tracks were generated with the GEMtools program v1.7.1 [46]. In addition, because the bin annotations that are included in the QDNAseq R package are based on 50 bp sequencing chemistry, we trimmed the 150 bp reads to 50 bp length using the crop utility of Trimmomatic v0.39 [47].

## Supplementary Information

Additional file 1: **Supplementary Figure 1**. Performance evaluation of the six copy number variation (CNV) algorithms using the simulated data with the loose criteria: at least 60% overlap between the inferred and ground truth CNV segments and inclusion of ≥ 0.5Mbp CNV segments. A) True positive rate(TPR), B) False discovery rate (FDR), and C) F1 score of the CNV detections achieved by the different tools when the read coverage is varied. The data points are based on the window size comparison results (**Supplementary Figures 2-6**), from which we selected the window settings that provided the highest F1 scores by the algorithms at each read coverage. Error bars denote the standard error of the results produced with 20 different random subsets. **Supplementary Figure 2**. Analysis of how the window size affects the performance of CNVnator at different read coverages with simulated data. A) True positive rate (TPR), B) False discovery rate (FDR), and C) F1 score. The hard criteria (minimum overlap of 0.8 and no filtering by size) were used in the analysis. **Supplementary Figure 3**. Analysis of how the window size affects the performance of BICseq2 at different read coverages with simulated data. A) True positive rate (TPR), B) False discovery rate (FDR), and C) F1 score. Default window size is 0.1 kbp. The hard criteria (minimum overlap of 0.8 and no filtering by size) were used in the analysis. **Supplementary Figure 4**. Analysis of how the window size affects the performance of FREEC at different read coverages with simulated data. A) True positive rate (TPR), B) False discovery rate (FDR), and C) F1 score. The coefficient of variation of 0.05 is the default value of the built-in method of FREEC for selecting the window size based on the coverage. The hard criteria (minimum overlap of 0.8 and no filtering by size) were used in the analysis. **Supplementary Figure 5**. Analysis of how the window size affects the performance of HMMcopy at different read coverages with simulated data. A) True positive rate (TPR), B) False discovery rate (FDR), and C) F1 score. The hard criteria (minimum overlap of 0.8 and no filtering by size) were used in the analysis. **Supplementary Figure 6**. Analysis of how the window size affects the performance of QDNAseq at different read coverages with simulated data. A) True positive rate (TPR), B) False discovery rate (FDR), and C) F1 score. The hard criteria (minimum overlap of 0.8 and no filtering by size) were used in the analysis. **Supplementary Figure 7**. Visualization of the CNVs detected in the H9-AB-p116 dataset using the six algorithms along with the array-based benchmark CNV segments in the respective chromosomal locations. Deletions are marked in red and gains in blue. The bottom part of the visualization depicts the depth of read coverage at each 50 kbp window. The visualization includes every CNV found with each tool using the window size that yielded the best performance for the simulated data at coverage of 0.1x (see **Supplementary Figures. 2-6**). **Supplementary Figure 8**. Visualization of the CNVs detected in the H9-AB-p113 dataset using the six algorithms along with the array-based benchmark CNV segments in the respective chromosomal locations. Deletions are marked in red and gains in blue. The bottom part of the visualization depicts the depth of read coverage at each 50 kbp window. The visualization includes every CNV found with each tool using the window size that yielded the best performance for the simulated data at coverage of 0.1x (see **Supplementary Figures. 2-6**). **Supplementary Figure 9**. Visualization of the CNVs detected in the H9-p38 dataset using the six algorithms along with the array-based benchmark CNV segments in the respective chromosomal locations. Deletions are marked in red and gains in blue. The bottom part of the visualization depicts the depth of read coverage at each 50 kbp window. The visualization includes every CNV found with each tool using the window size that yielded the best performance for the simulated data at coverage of 0.1x (see **Supplementary Figures**. 2-6). **Supplementary Figure 10**. Visualization of the CNVs detected in the H9-p41 dataset using the six algorithms along with the array-based benchmark CNV segments in the respective chromosomal locations. Deletions are marked in red and gains in blue. The bottom part of the visualization depicts the depth of read coverage at each 50 kbp window. The visualization includes every CNV found with each tool using the window size that yielded the best performance for the simulated data at coverage of 0.1x (see **Supplementary Figures. 2-6**). **Supplementary Figure 11**. Visualization of the CNVs detected in all the chromosomes in the combined sample H9-AB by the six algorithms along with the array-based benchmark CNV segments in the respective chromosomal locations. Deletions are marked in red and gains in blue. The bottompart of the visualization depicts the depth of read coverage at each 50 kbp window. The visualizationincludes every CNV found with each tool using the window size that yielded the best performancefor the simulated data at coverage of 0.1x (see **Supplementary Figures. 2-6**). **Supplementary Figure 12**. Visualization of the CNVs detected in all the chromosomes in the combined sample H9-NO by the six algorithms along with the array-based benchmark CNV segments in the respective chromosomal locations. Deletions are marked in red and gains in blue. The bottom part of the visualization depicts the depth of read coverage at each 50 kbp window. The visualization includes every CNV found with each tool using the window size that yielded the best performance for the simulated data at coverage of 0.1x (see **Supplementary Figs. 2-6**). **Supplementary Figure 13. ** Visualization of the CNVs detected in all the chromosomes in the combined sample H9-AB-p116 by the six algorithms along with the array-based benchmark CNV segments in the respective chromosomal locations. Deletions are marked in red and gains in blue. The bottom part of the visualization depicts the depth of read coverage at each 50 kbp window. The visualization includes every CNV found with each tool using the window size that yielded the best performance for the simulated data at coverage of 0.1x. **Supplementary Figure 14**. Visualization of the CNVs detected in all the chromosomes in the combined sample H9-AB-p113 by the six algorithms along with the array-based benchmark CNV segments in the respective chromosomal locations. Deletions are marked in red and gains in blue. The bottom part of the visualization depicts the depth of read coverage at each 50 kbp window. The visualization includes every CNV found with each tool using the window size that yielded the best performance for the simulated data at coverage of 0.1x (see **Supplementary Figs. 2-6**). **Supplementary Figure 15**. Visualization of the CNVs detected in all the chromosomes in the combined sample H9-NO-p41 by the six algorithms along with the array-based benchmark CNV segments in the respective chromosomal locations. Deletions are marked in red and gains in blue. The bottom part of the visualization depicts the depth of read coverage at each 50 kbp window. The visualization includes every CNV found with each tool using the window size that yielded the best performance for the simulated data at coverage of 0.1x (see **Supplementary Figs. 2-6**). **Supplementary Figure 16**. Visualization of the CNVs detected in all the chromosomes in the combined sample H9-NO-p38 by the six algorithms along with the array-based benchmark CNV segments in the respective chromosomal locations. Deletions are marked in red and gains in blue. The bottom part of the visualization depicts the depth of read coverage at each 50 kbp window. All chromosomes included. Combined sample H9-NO-p38. The visualization includes every CNV found with each tool using the window size that yielded the best performance for the simulated data at coverage of 0.1x (see **Supplementary Figs. 2-6**). **Supplementary Figure 17**. Performance evaluation of the six algorithms using the cell line data with the stringent criteria: at least 80% overlap between the inferred and array-validated CNV segments and ≥0.5Mbp CNV length requirement for the detected CNV segment. A,D) True positive rate, B,E) False discovery rate and C,F) F1 score of the CNV detections. The red and blue dots depict the abnormal and normal samples, respectively. With each tool we used the the window size that yielded the best performance for the simulated data at coverage of 0.1x (see **Supplementary Figs. 2-6**). **Supplementary Table 1**. Simulated CNV segments that were used to evaluate the tools. **Supplementary Table 2**. Number of bases and read coverage of the cell line samples for each sample individually and for the combined samples. **Supplementary Table 3**. Array-based CNV segments ≥500 kbp used to evaluate the tools. **Supplementary Table 4**. Failure rates for different read coverages with varying window size settings and 20 different down samplings using simulated data.

## Data Availability

The human pluripotent embryonic stem cell line H9 raw sequence files are available in the NCBI repository (https://www.ncbi.nlm.nih.gov/bioproject/726033) with accession number PRJNA726033. The ground truth CNVs as well as the raw simulated data are available in the Zenodo repository (https://zenodo.org/record/4727293#.YIq0FGhRW-y). The ground truth CNVs accompanying the simulated dataset can be found in Supplementary Table 1.

## Abbreviations

BAMBAM: Binary alignment map
BIC: Bayesian information criterion
CBS: Circular binary segmentation
CNV: Copy number variation
**FDR**: False discovery rate
FFPE: Formalin-fixed paraffin embedded
hESC: Human embryonic stem cell
HMM: Hidden Markov model
LASSO: Least absolute shrinkage and selection operator
LOH: Loss of heterozygosity
TPR: True positive rate
WES: Whole-exome sequencing
WGS: Whole-genome sequencing
