# Supplementary material for: Evaluation of tools for identifying large copy number variations from ultra-low-coverage whole-genome sequencing data
Source: BMC Genomics. 2021 May 17;22:357. doi: 10.1186/s12864-021-07686-z (PMC8130438; doi:10.1186/s12864-021-07686-z)
Supplement: Supplementary file 1 — Supplementary Figure 1. Performance evaluation of the six copy number variation (CNV) algorithms using the simulated data with the loose criteria: at least 60% overlap between the inferred and ground truth CNV segments and inclusion of ≥ 0.5Mbp CNV segments. A) True positive rate(TPR), B) False discovery rate (FDR), and C) F1 score of the CNV detections achieved by the different tools when the read coverage is varied. The data points are based on the window size comparison results (Supplementary Figures 2-6), from which we selected the window settings that provided the highest F1 scores by the algorithms at each read coverage. Error bars denote the standard error of the results produced with 20 different random subsets. Supplementary Figure 2. Analysis of how the window size affects the performance of CNVnator at different read coverages with simulated data. A) True positive rate (TPR), B) False discovery rate (FDR), and C) F1 score. The hard criteria (minimum overlap of 0.8 and no filtering by size) were used in the analysis. Supplementary Figure 3. Analysis of how the window size affects the performance of BICseq2 at different read coverages with simulated data. A) True positive rate (TPR), B) False discovery rate (FDR), and C) F1 score. Default window size is 0.1 kbp. The hard criteria (minimum overlap of 0.8 and no filtering by size) were used in the analysis. Supplementary Figure 4. Analysis of how the window size affects the performance of FREEC at different read coverages with simulated data. A) True positive rate (TPR), B) False discovery rate (FDR), and C) F1 score. The coefficient of variation of 0.05 is the default value of the built-in method of FREEC for selecting the window size based on the coverage. The hard criteria (minimum overlap of 0.8 and no filtering by size) were used in the analysis. Supplementary Figure 5. Analysis of how the window size affects the performance of HMMcopy at different read coverages with simulated data. A) True positive rat [file 12864_2021_7686_MOESM1_ESM.pdf]

Supplementary Figures and Tables

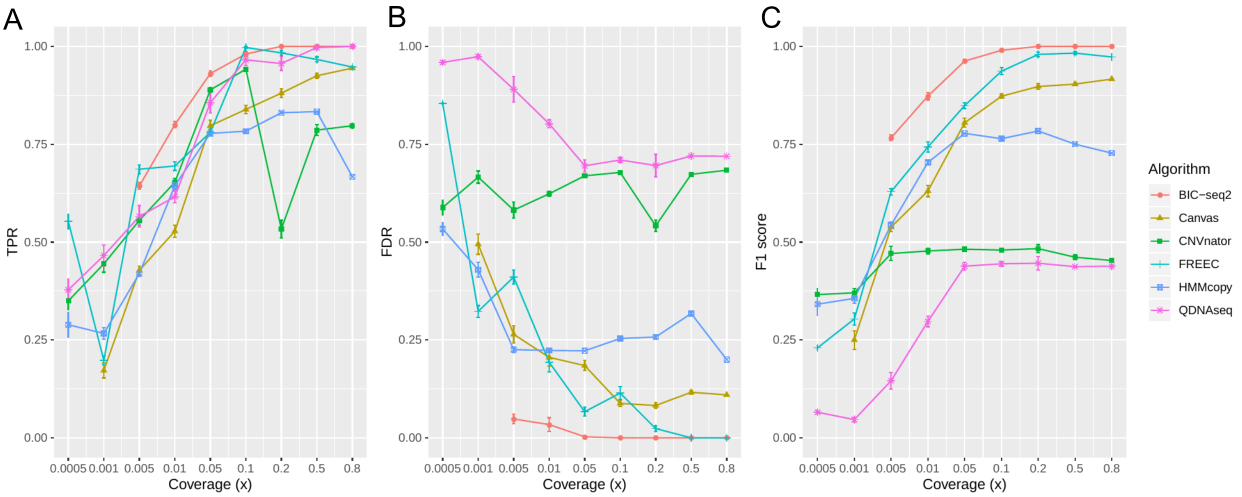

Supplementary Figure 1. Performance evaluation of the six copy number variation (CNV) algorithms using the simulated data with the loose criteria: at least 60% overlap between the inferred and ground truth CNV segments and inclusion of ≥ 0.5Mbp CNV segments. A) True positive rate (TPR), B) False discovery rate (FDR), and C) F1 score of the CNV detections achieved by the different tools when the read coverage is varied. The data points are based on the window size comparison results (Supplementary Figures 2-6), from which we selected the window settings that provided the highest F1 scores by the algorithms at each read coverage. Error bars denote the standard error of the results produced with 20 different random subsets.

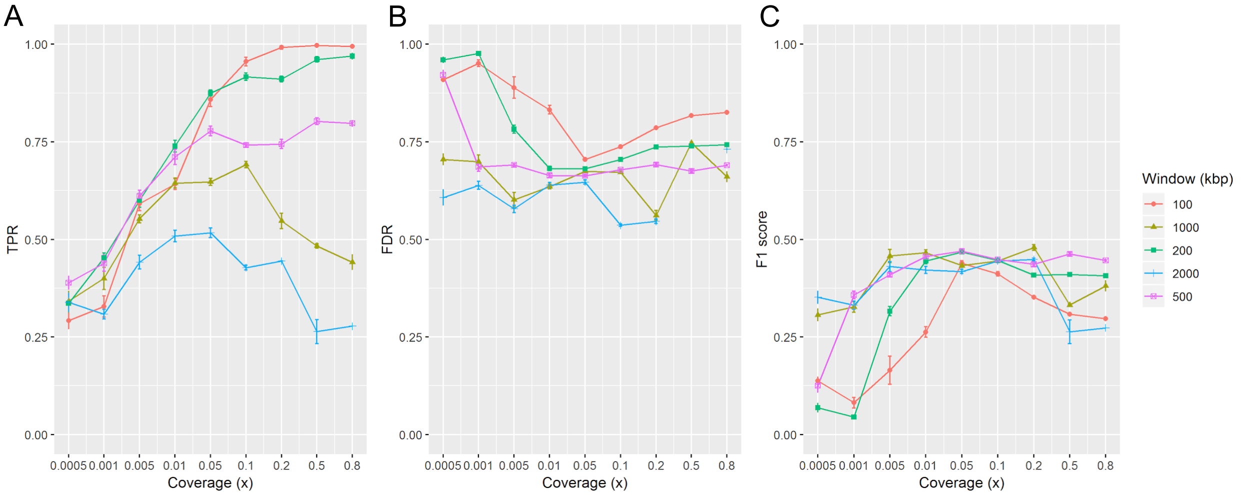

Supplementary Figure 2. Analysis of how the window size affects the performance of CNVnator at different read coverages with simulated data. A) True positive rate (TPR), B) False discovery rate (FDR), and C) F1 score. The hard criteria (minimum overlap of 0.8 and no filtering by size) were used in the analysis.

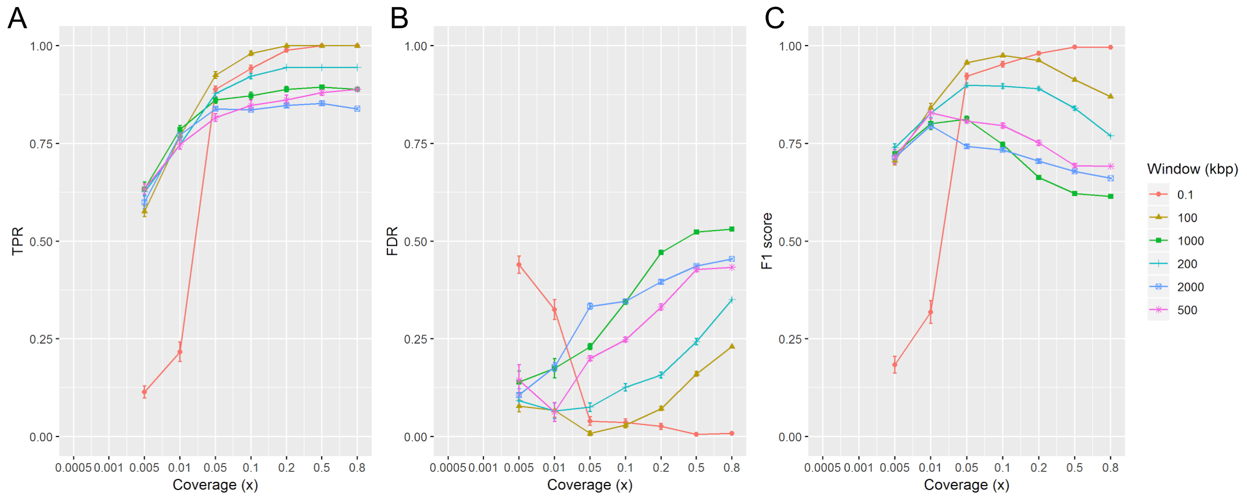

Supplementary Figure 3. Analysis of how the window size affects the performance of BICseq2 at different read coverages with simulated data. A) True positive rate (TPR), B) False discovery rate (FDR), and C) F1 score. Default window size is 0.1 kbp. The hard criteria (minimum overlap of 0.8 and no filtering by size) were used in the analysis.

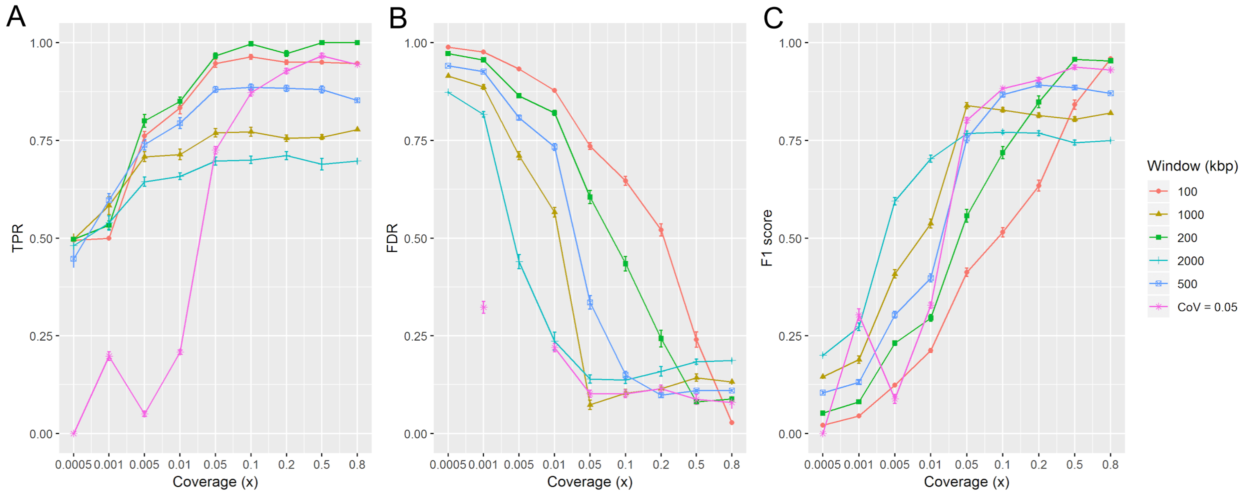

Supplementary Figure 4. Analysis of how the window size affects the performance of FREEC at different read coverages with simulated data. A) True positive rate (TPR), B) False discovery rate (FDR), and C) F1 score. The coefficient of variation of 0.05 is the default value of the built-in method of FREEC for selecting the window size based on the coverage. The hard criteria (minimum overlap of 0.8 and no filtering by size) were used in the analysis.

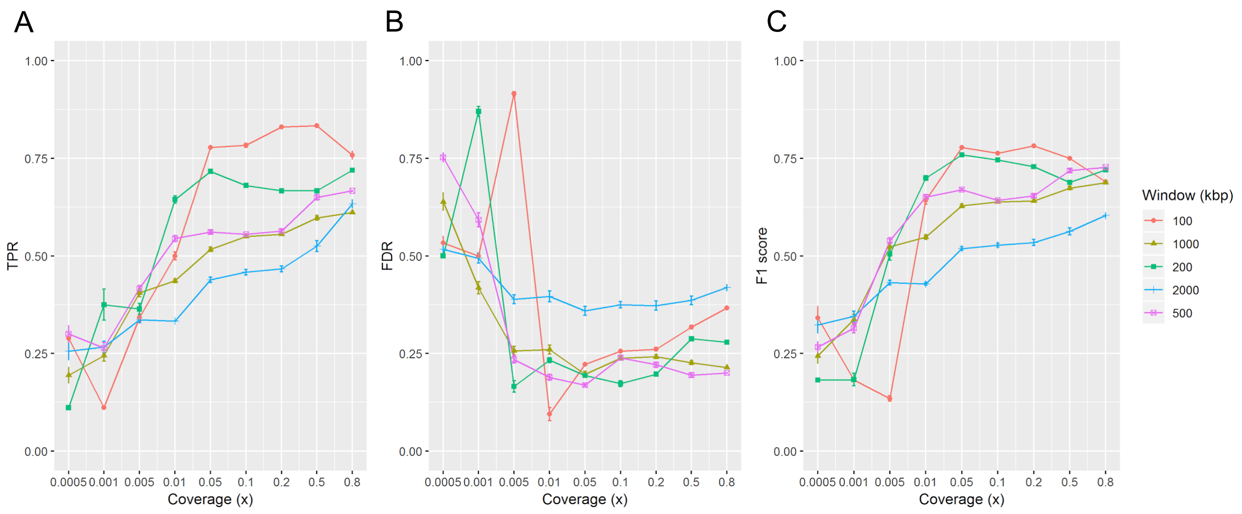

Supplementary Figure 5. Analysis of how the window size affects the performance of HMMcopy at different read coverages with simulated data. A) True positive rate (TPR), B) False discovery rate (FDR), and C) F1 score. The hard criteria (minimum overlap of 0.8 and no filtering by size) were used in the analysis.

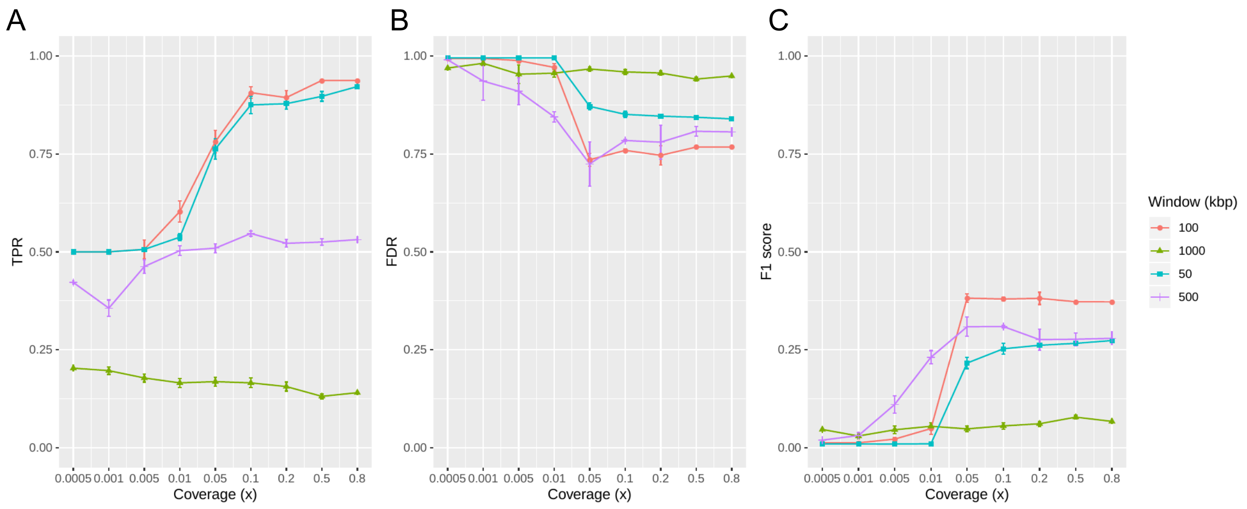

Supplementary Figure 6. Analysis of how the window size affects the performance of QDNAseq at different read coverages with simulated data. A) True positive rate (TPR), B) False discovery rate (FDR), and C) F1 score. The hard criteria (minimum overlap of 0.8 and no filtering by size) were used in the analysis.

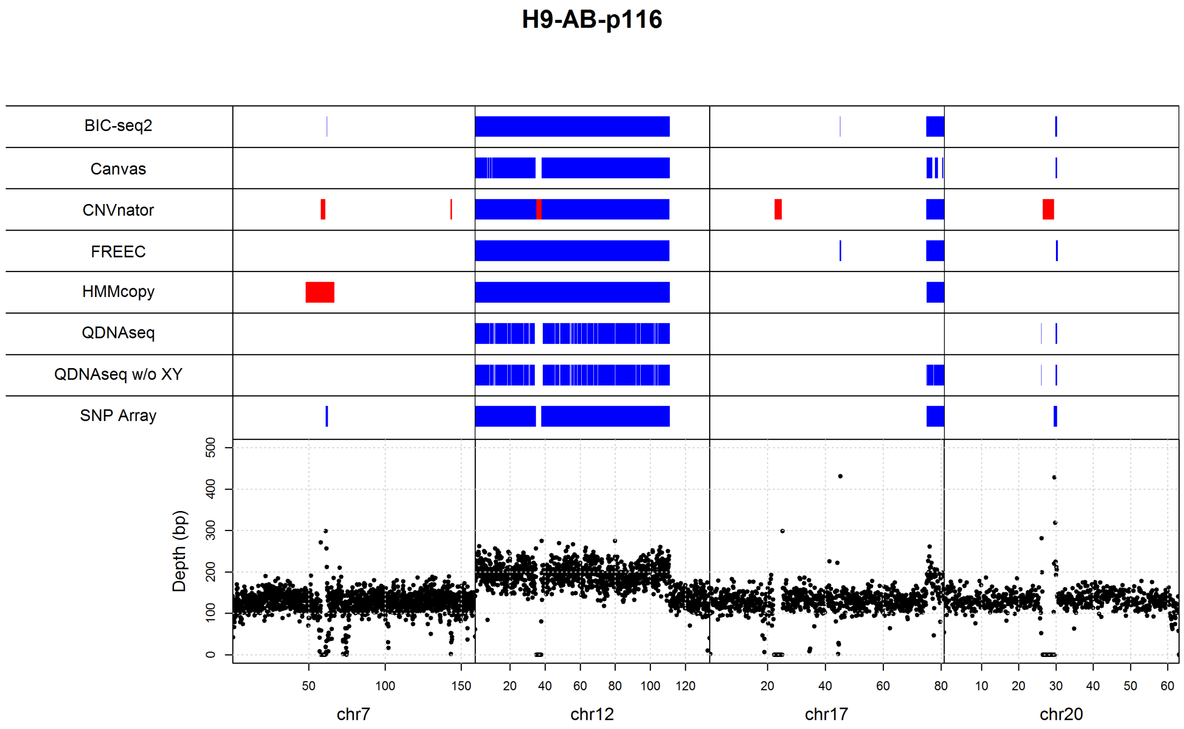

Supplementary Figure 7. Visualization of the CNVs detected in the H9-AB-p116 dataset using the six algorithms along with the array-based benchmark CNV segments in the respective chromosomal locations. Deletions are marked in red and gains in blue. The bottom part of the visualization depicts the depth of read coverage at each 50 kbp window. The visualization includes every CNV found with each tool using the window size that yielded the best performance for the simulated data at coverage of 0.1x (see Supplementary Figs. 2-6).

H9-AB-p113

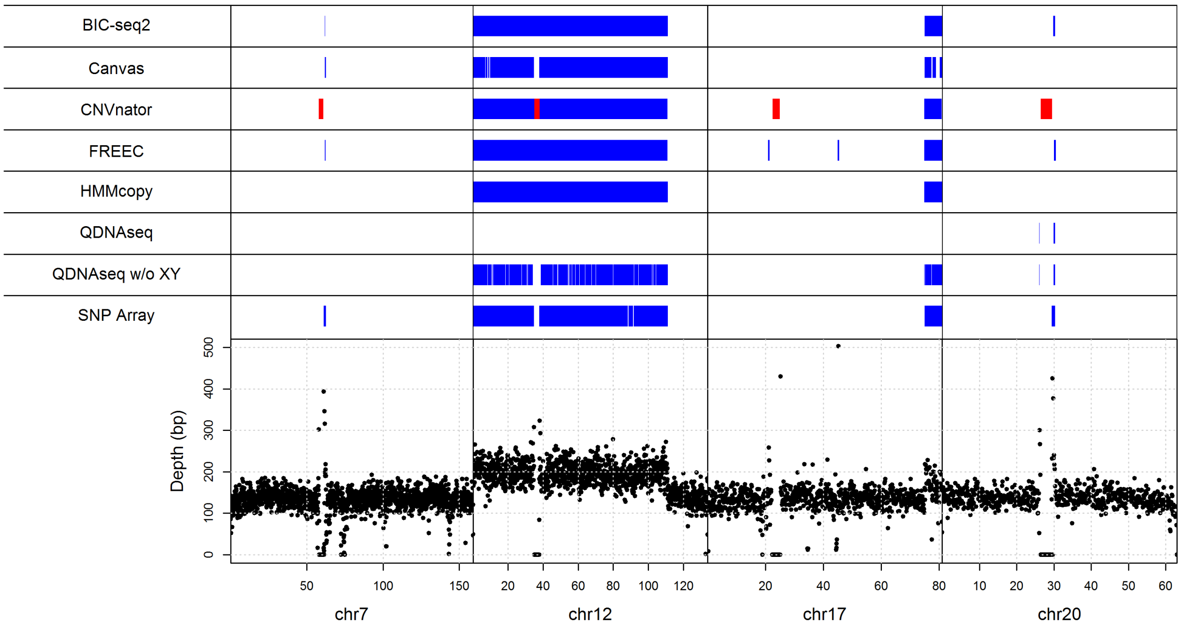

Supplementary Figure 8. Visualization of the CNVs detected in the H9-AB-p113 dataset using the six algorithms along with the array-based benchmark CNV segments in the respective chromosomal locations. Deletions are marked in red and gains in blue. The bottom part of the visualization depicts the depth of read coverage at each 50 kbp window. The visualization includes every CNV found with each tool using the window size that yielded the best performance for the simulated data at coverage of 0.1x (see Supplementary Figs. 2-6).

H9-p38

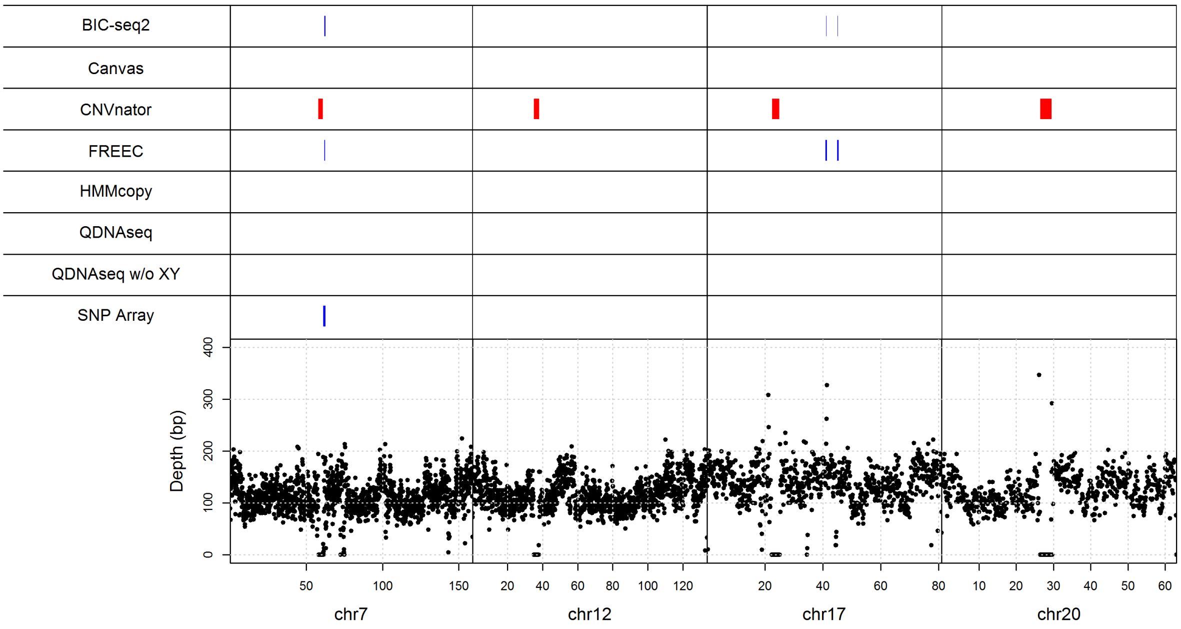

Supplementary Figure 9. Visualization of the CNVs detected in the H9-p38 dataset using the six algorithms along with the array-based benchmark CNV segments in the respective chromosomal locations. Deletions are marked in red and gains in blue. The bottom part of the visualization depicts the depth of read coverage at each 50 kbp window. The visualization includes every CNV found with each tool using the window size that yielded the best performance for the simulated data at coverage of 0.1x (see Supplementary Figs. 2-6).

H9-p41

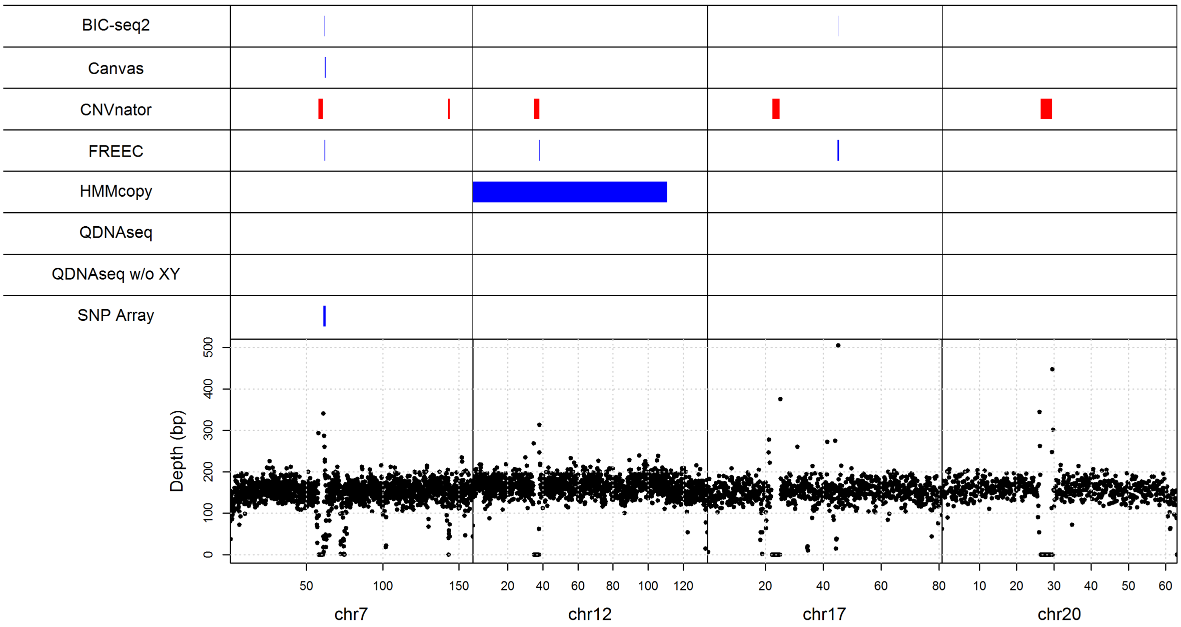

Supplementary Figure 10. Visualization of the CNVs detected in the H9-p41 dataset using the six algorithms along with the array-based benchmark CNV segments in the respective chromosomal locations. Deletions are marked in red and gains in blue. The bottom part of the visualization depicts the depth of read coverage at each 50 kbp window. The visualization includes every CNV found with each tool using the window size that yielded the best performance for the simulated data at coverage of 0.1x (see Supplementary Figs. 2-6).

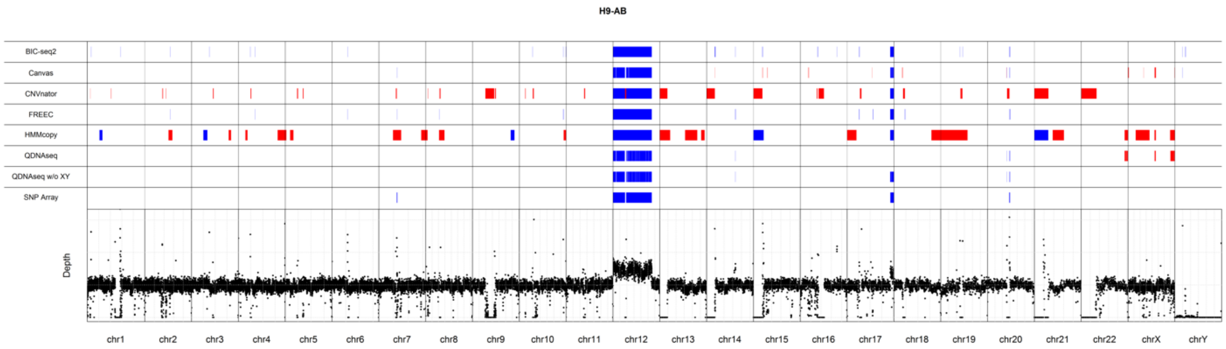

Supplementary Figure 11. Visualization of the CNVs detected in all the chromosomes in the combined sample H9-AB by the six algorithms along with the array-based benchmark CNV segments in the respective chromosomal locations. Deletions are marked in red and gains in blue. The bottom part of the visualization depicts the depth of read coverage at each 50 kbp window. The visualization includes every CNV found with each tool using the window size that yielded the best performance for the simulated data at coverage of 0.1x (see Supplementary Figs. 2-6).

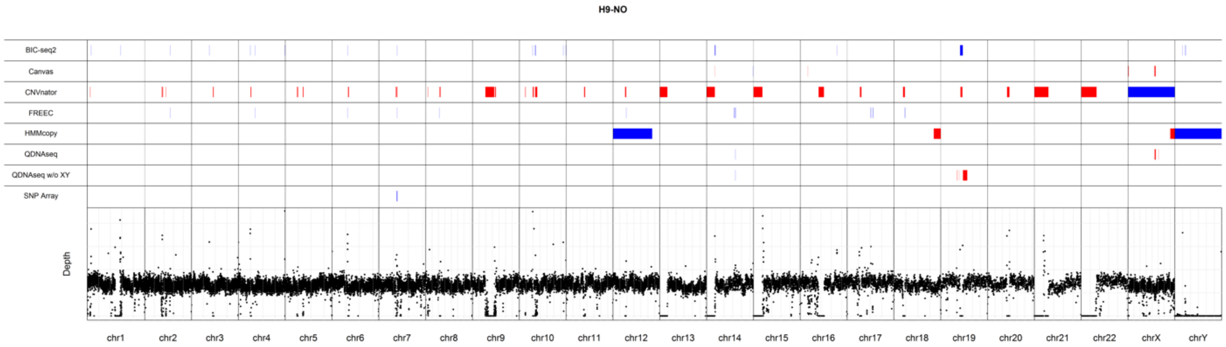

Supplementary Figure 12. Visualization of the CNVs detected in all the chromosomes in the combined sample H9-NO by the six algorithms along with the array-based benchmark CNV segments in the respective chromosomal locations. Deletions are marked in red and gains in blue. The bottom part of the visualization depicts the depth of read coverage at each 50 kbp window. The visualization includes every CNV found with each tool using the window size that yielded the best performance for the simulated data at coverage of 0.1x (see Supplementary Figs. 2-6).

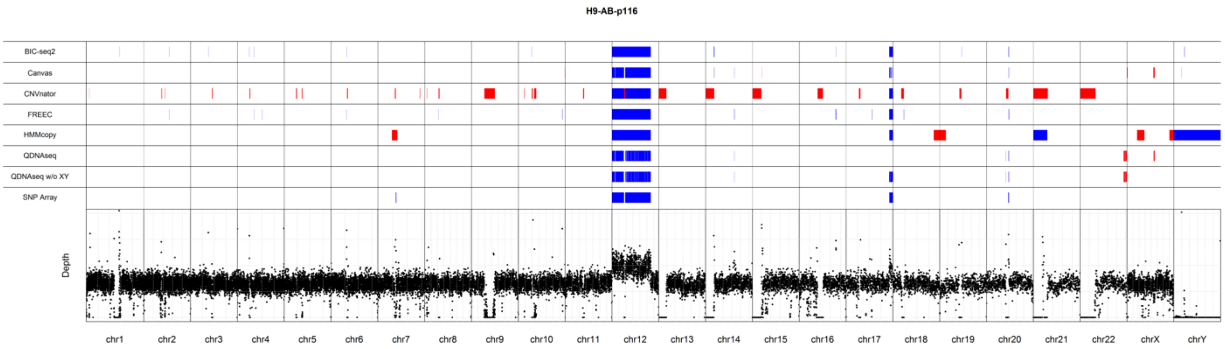

Supplementary Figure 13. Visualization of the CNVs detected in all the chromosomes in the combined sample H9-AB-p116 by the six algorithms along with the array-based benchmark CNV segments in the respective chromosomal locations. Deletions are marked in red and gains in blue. The bottom part of the visualization depicts the depth of read coverage at each 50 kbp window. The visualization includes every CNV found with each tool using the window size that yielded the best performance for the simulated data at coverage of 0.1x.

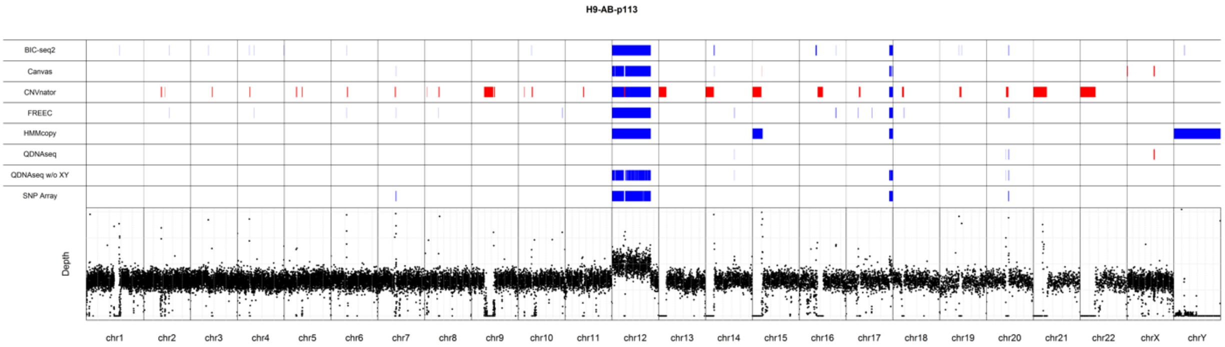

Supplementary Figure 14. Visualization of the CNVs detected in all the chromosomes in the combined sample H9-AB-p113 by the six algorithms along with the array-based benchmark CNV segments in the respective chromosomal locations. Deletions are marked in red and gains in blue. The bottom part of the visualization depicts the depth of read coverage at each 50 kbp window. The visualization includes every CNV found with each tool using the window size that yielded the best performance for the simulated data at coverage of 0.1x (see Supplementary Figs. 2-6).

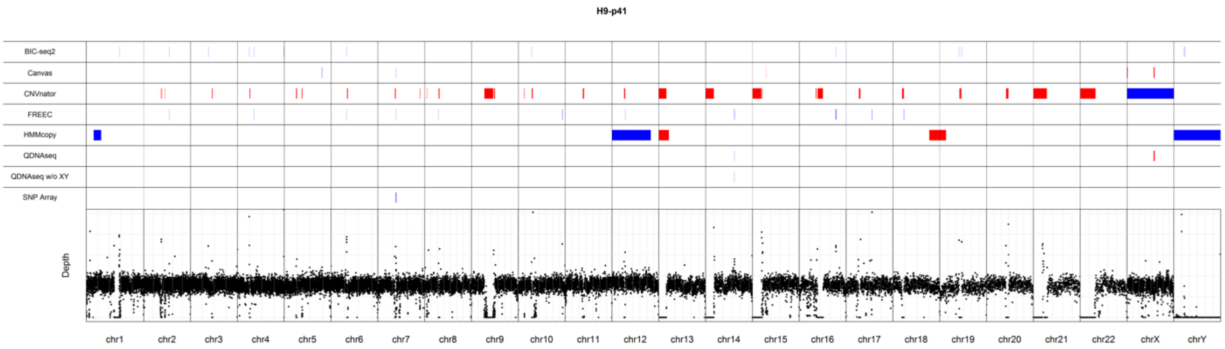

Supplementary Figure 15. Visualization of the CNVs detected in all the chromosomes in the combined sample H9-NO-p41 by the six algorithms along with the array-based benchmark CNV segments in the respective chromosomal locations. Deletions are marked in red and gains in blue. The bottom part of the visualization depicts the depth of read coverage at each 50 kbp window. The visualization includes every CNV found with each tool using the window size that yielded the best performance for the simulated data at coverage of 0.1x (see Supplementary Figs. 2-6).

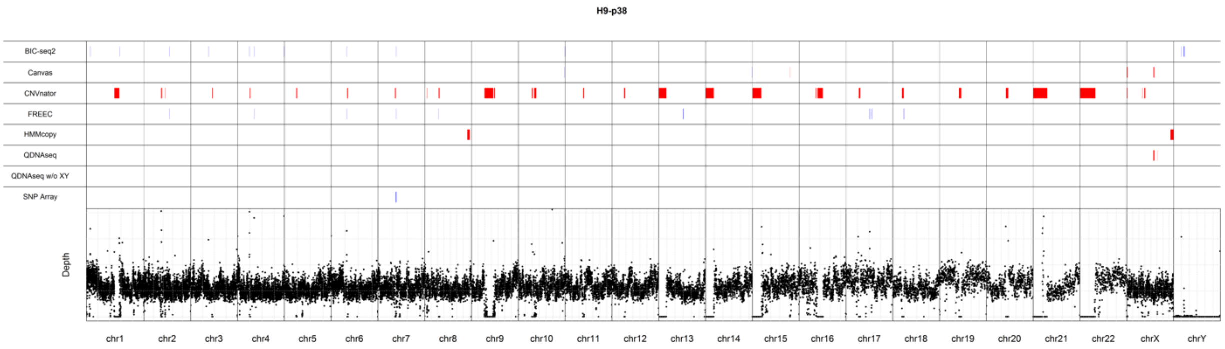

Supplementary Figure 16. Visualization of the CNVs detected in all the chromosomes in the combined sample H9-NO-p38 by the six algorithms along with the array-based benchmark CNV segments in the respective chromosomal locations. Deletions are marked in red and gains in blue. The bottom part of the visualization depicts the depth of read coverage at each 50 kbp window. All chromosomes included. Combined sample H9-NO-p38. The visualization includes every CNV found with each tool using the window size that yielded the best performance for the simulated data at coverage of 0.1x (see Supplementary Figs. 2-6).

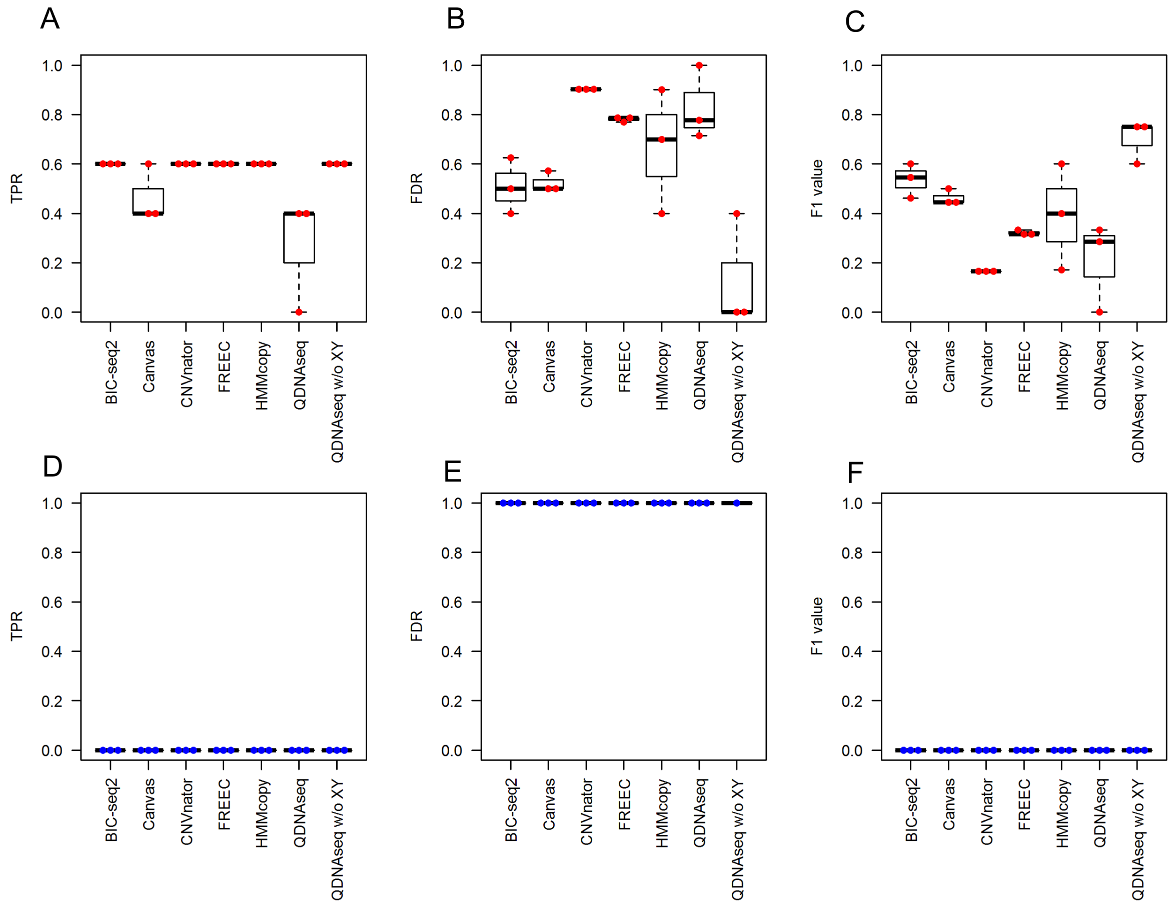

Supplementary Figure 17. Performance evaluation of the six algorithms using the cell line data with the stringent criteria: at least 80% overlap between the inferred and array-validated CNV segments and ≥0.5Mbp CNV length requirement for the detected CNV segment. A,D) True positive rate, B,E) False discovery rate and C,F) F1 score of the CNV detections. The red and blue dots depict the abnormal and normal samples, respectively. With each tool we used the the window size that yielded the best performance for the simulated data at coverage of 0.1x (see Supplementary Figs. 2-6).

Supplementary Table 1. Simulated CNV segments that were used to evaluate the tools.

Chromosome	Start	End	Length (Mbp)	Copy number
chr2	162543986	212543985	50	4
chr3	25351596	30351595	5	4
chr3	95201231	96201230	1	3
chr3	132457135	162457134	30	4
chr5	51493737	53493736	2	1
chr5	55182578	75182577	20	1
chr5	105754266	135754265	30	0
chr6	80631176	81631175	1	1
chr6	103969646	113969645	10	1
chr8	91996547	101996546	10	3
chr10	14963021	16963020	2	4
chr10	64735169	114735168	50	1
chr15	92718330	97718329	5	0
chr16	11746483	31746482	20	4
chrX	83875728	88875727	5	0
chrX	132082431	137082430	5	2
chrY	2941997	7941996	5	0
chrY	14336476	19336475	5	3

Supplementary Table 2. Number of bases and read coverage of the cell line samples for each sample individually and for the combined samples.

Sample	Number of bases	Read coverage (x)
H9-AB-p113, rep1	321241682	0,11
H9-AB-p113, rep2	240670548	0,08
H9-AB-p113, rep3	192887266	0,06
H9-AB-p113, rep4	179233578	0,06
H9-AB-p116, rep1	294468864	0,10
H9-AB-p116, rep2	236160272	0,08
H9-AB-p116, rep3	218629920	0,07
H9-AB-p116, rep4	330354402	0,11
H9-NO-p38, rep1	277405116	0,09
H9-NO-p38, rep2	195872716	0,07
H9-NO-p41, rep1	381214876	0,13
H9-NO-p41, rep2	238732380	0,08
H9-AB-p113 (1+2)[a]	561912230	0,19
H9-AB-p116 (1+2)[a]	530629136	0,18
H9-NO-p38 (1+2)[a]	473277832	0,16
H9-NO-p41 (1+2)[a]	619947256	0,21
H9-AB[b]	2013646532	0,67
H9-NO[b]	1093225088	0,36

[a]Replicates 1 and 2 were merged

[b]All the eight replicate samples for H9-AB and four replicate samples for H9-NO were combined as one sample, respectively.

Supplementary Table 3. Array-based CNV segments ≥500 kbp used to evaluate the tools.

Sample	CNV (Chr:Start:End:Copy number value)
H9-AB-p113	chr7:61056053:62699114:3 chr12:191619:34801271:3 chr12:37927114:88375488:3 chr12:88658684:91296986:3 chr12:91652678:111158848:3 chr17:75004213:81052262:3 chr20:29419620:30365359:3
H9-AB-p116	chr7:61056053:62689447:3 chr12:191619:34801271:3 chr12:37927114:111100677:3 chr17:75022811:81052262:3 chr20:29419620:30365359:3
H9-NO-p38	chr7:61056053:62699114:3
H9-NO-p41	chr7:61056053:62699114:3

Supplementary Table 4. Failure rates for different read coverages with varying window size settings and 20 different downsamplings using simulated data.

**Algorithm/ Coverage**	**0.0005**	**0.001**	**0.005**	**0.01**	**0.05**	**0.1**	**0.2**	**0.5**	**0.8**
BIC-seq2	1	1	0.0167	0	0	0	0	0	0
Canvas	1	0	0	0	0	0	0	0	0
CNVnator	0	0	0	0	0	0	0	0	0
FREEC	0.1583	0.033	0	0	0	0	0	0	0
HMMcopy	0	0	0	0	0	0	0	0	0
QDNAseq	0	0	0	0	0	0	0	0	0
